# Dichlorido(6,6′-dimethyl-2,2′-bipyridine-κ^2^ *N*,*N*′)zinc(II)

**DOI:** 10.1107/S1600536809038215

**Published:** 2009-09-26

**Authors:** Robabeh Alizadeh, Khadijeh Kalateh, Amin Ebadi, Roya Ahmadi, Vahid Amani

**Affiliations:** a Damghan University of Basic Sciences, School of Chemistry, Damghan, Iran; b Islamic Azad University, Shahr-e-Rey Branch, Tehran, Iran; c Department of Chemistry, Islamic Azad University, Kazerun Branch, Kazerun, Fars, Iran

## Abstract

In the title compound, [ZnCl_2_(C_12_H_12_N_2_)], the complete mol­ecule is generated by crystallographic mirror symmetry, with the Zn atom and both chloride ions lying on the reflecting plane, yielding a distorted ZnN_2_Cl_2_ tetra­hedral coordination for the metal ion. In the crystal, there are π–π contacts between the pyridine rings [centroid–centroid distance = 3.7857 (17) Å].

## Related literature

For related structures containing Zn bonded to two chloride ions and a phenanthroline/bipyridine derivative, see: Ahmadi *et al.* (2008 ▶, 2009*a* ▶,*b* ▶); Alizadeh *et al.* (2009 ▶); Gruia *et al.* (2007 ▶); Khalighi *et al.* (2008 ▶); Khan & Tuck (1984 ▶); Khavasi *et al.* (2008 ▶); Khoshtarkib *et al.* (2009 ▶); Kozhevnikov *et al.* (2006 ▶); Liu *et al.* (2004 ▶); Preston & Kennard (1969 ▶); Reimann *et al.* (1966 ▶).
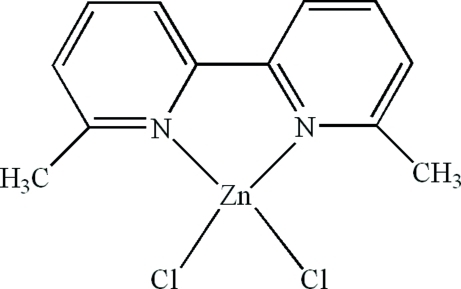


## Experimental

### 

#### Crystal data

[ZnCl_2_(C_12_H_12_N_2_)]
*M* *_r_* = 320.53
Monoclinic, 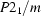

*a* = 7.6957 (15) Å
*b* = 11.266 (2) Å
*c* = 8.1431 (16) Å
β = 110.61 (3)°
*V* = 660.8 (3) Å^3^
*Z* = 2
Mo *K*α radiation
μ = 2.24 mm^−1^
*T* = 298 K
0.40 × 0.33 × 0.30 mm

#### Data collection

Bruker SMART CCD diffractometer
Absorption correction: multi-scan (*SADABS*; Sheldrick, 1998 ▶) *T*_min_ = 0.421, *T*_max_ = 0.512
8852 measured reflections
2075 independent reflections
1972 reflections with *I* > 2σ(*I*)
*R*_int_ = 0.043

#### Refinement

*R*[*F* ^2^ > 2σ(*F* ^2^)] = 0.034
*wR*(*F* ^2^) = 0.101
*S* = 1.26
2075 reflections
83 parameters
H-atom parameters constrained
Δρ_max_ = 0.70 e Å^−3^
Δρ_min_ = −0.55 e Å^−3^

### 

Data collection: *SMART* (Bruker, 1998 ▶); cell refinement: *SAINT* (Bruker, 1998 ▶); data reduction: *SAINT*; program(s) used to solve structure: *SHELXTL* (Sheldrick, 2008 ▶); program(s) used to refine structure: *SHELXTL*; molecular graphics: *ORTEP-3* (Farrugia, 1997 ▶); software used to prepare material for publication: *WinGX* (Farrugia, 1999 ▶).

## Supplementary Material

Crystal structure: contains datablocks I, global. DOI: 10.1107/S1600536809038215/hb5106sup1.cif

Structure factors: contains datablocks I. DOI: 10.1107/S1600536809038215/hb5106Isup2.hkl

Additional supplementary materials:  crystallographic information; 3D view; checkCIF report

## Figures and Tables

**Table:** 

Zn1—N1	2.0569 (18)
Zn1—Cl1	2.2013 (11)
Zn1—Cl2	2.2035 (10)

**Table:** 

N1^i^—Zn1—N1	80.71 (10)

Symmetry code: (i) 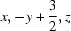.

**Fig. 1.**
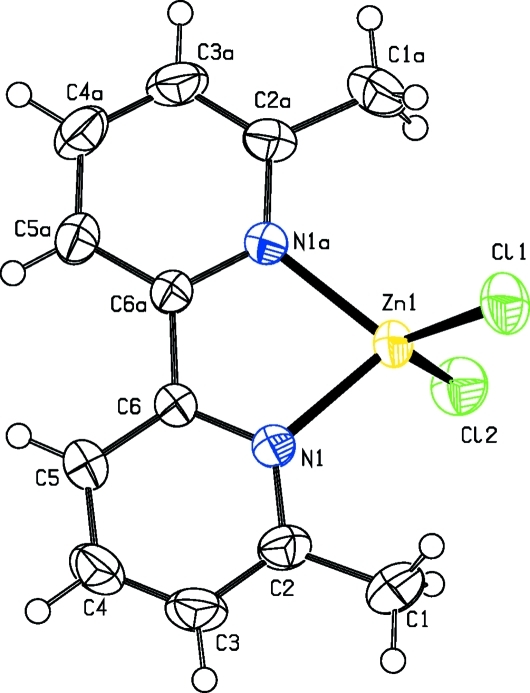
The molecular structure of (I). Displacement ellipsoids are drawn at the 50% probability level. [Symmetry codes: (a) x,-y + 3/2,z]

**Fig. 2.**
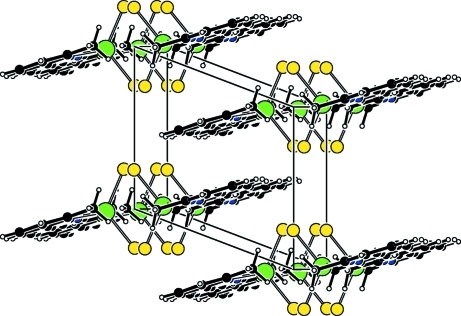
Tha unit-cell packing of (I).

## supplementary crystallographic information

### Comment

Recently, we reported the synthes and crystal structure of [ZnCl_2_(phend)], (II), (Khoshtarkib *et al.*, 2009), [HgBr_2_(2,9-dmphen)], (III), (Alizadeh *et al.*, 2009), [HgCl_2_(2,9-dmPh2phen)].0.5 CH_3_CN, (IV) (Ahmadi, *et al.*, 2009*a*) and [Pb_4_(NO_3_)_8_(6-mbpy)_4_], (V), (Ahmadi, *et al.*, 2009*b*) [where phend is phenanthridine, 2,9-dmphen is 2,9-dimethyl-1,10-phenanthroline, 2,9-dmPh2phen is 2,9-dimethyl-4,7-diphenyl-1,10-phenanthroline and 6-mbpy is 6-methyl-2,2'-bipyridine].

There are several Zn^II^ complexes, with formula, [ZnCl_2_(N—N)], such as [ZnCl_2_(bipy)], (VI), (Khan & Tuck, 1984), [ZnCl_2_(biim)], (VII), (Gruia *et al.*, 2007), [ZnCl_2_(phbipy)], (IIX), (Kozhevnikov *et al.*, 2006), [ZnCl_2_(phen)], (IX), (Reimann *et al.*, 1966), [ZnCl_2_(dmphen)], (*X*), (Preston & Kennard, 1969), [ZnCl_2_(dpdmbip)], (XI), (Liu *et al.*, 2004), [ZnCl_2_(dm4bt)], (XII), (Khavasi *et al.*, 2008), [ZnCl_2_(5,5'-dmbpy)], (XIII), (Khalighi *et al.*, 2008) and [ZnCl_2_(6-mbpy)], (XIV), (Ahmadi, Kalateh, Ebadi *et al.*, 2008) [where bipy is 2,2'-bipyridine, biim is 2,2'-biimidazole, phbipy is 5-phenyl-2,2'-bipyridine, phen is 1,10-phenanthroline, dmphen is 2,9-dimethyl-1,10-phenanthroline, dpdmbip is 4,4'-diphenyl-6,6'-dimethyl-2,2'-bipyrimidine, dm4bt is 2,2'-dimethyl-4,4'-bithiazole and 5,5'-dmbpy 5,5'-dimethyl-2,2'-bipyridine] have been synthesized and characterized by single-crystal X-ray diffraction methods. We report herein the synthesis and crystal structure of the title compound (I).

The asymmetric unit of the title compound, (I), (Fig. 1), contains half molecule. The Zn^II^ atom is four-coordinated in distorted tetrahedral configurations by two N atoms from one 6,6'-dimethyl-2,2'-bipyridine and two terminal Cl atoms. The Zn—Cl and Zn—N bond lengths and angles are collected in Table 1.

In the crystal structure, the π-π contacts between the rings A (N1/C2—C6) and rings A, *Cg*2···*Cg*2^i^ [distance = 3.7857 (17) Å, symmetry cods: 1-*X*,2-Y,1-*Z*]. It seems this π-π stacking is effective in the stabilization of the crystal structure (Fig. 2).

### Experimental

A solution of 6,6'-dimethyl-2,2'-bipyridine (0.20 g, 1.10 mmol) in methanol (10 ml) was added to a solution of ZnCl_2_ (0.15 g, 0.88 mmol) in acetonitrile (10 ml) and the resulting colourless solution was stirred for 20 min at at 313 K. This solution was left to evaporate slowly at room temperature. After one week, colorless prisms of (I) were isolated (yield 0.26 g, 73.7%).

### Refinement

All H atoms were positioned geometrically, with C—H = 0.93–0.96Å and constrained to ride on their parent atoms, with *U*_iso_(H)=1.2*U*_eq_(C) or 1.5U_eq_(methyl C).

### Crystal data

**Table:**

[ZnCl_2_(C_12_H_12_N_2_)]	*F*(000) = 324
*M**_r_* = 320.53	*D*_x_ = 1.611 Mg m^−^^3^
Monoclinic, *P*2_1_/*m*	Mo *K*α radiation, λ = 0.71073 Å
Hall symbol: -P 2yb	Cell parameters from 1170 reflections
*a* = 7.6957 (15) Å	θ = 2.8–30.6°
*b* = 11.266 (2) Å	µ = 2.24 mm^−^^1^
*c* = 8.1431 (16) Å	*T* = 298 K
β = 110.61 (3)°	Prism, colourless
*V* = 660.8 (3) Å^3^	0.40 × 0.33 × 0.30 mm
*Z* = 2	

### Data collection

**Table:**

Bruker SMART CCD diffractometer	2075 independent reflections
Radiation source: fine-focus sealed tube	1972 reflections with *I* > 2σ(*I*)
graphite	*R*_int_ = 0.043
ω scans	θ_max_ = 30.6°, θ_min_ = 2.8°
Absorption correction: multi-scan (*SADABS*; Sheldrick, 1998)	*h* = −10→10
*T*_min_ = 0.421, *T*_max_ = 0.512	*k* = −16→16
8852 measured reflections	*l* = −11→10

### Refinement

**Table:**

Refinement on *F*^2^	Primary atom site location: structure-invariant direct methods
Least-squares matrix: full	Secondary atom site location: difference Fourier map
*R*[*F*^2^ > 2σ(*F*^2^)] = 0.034	Hydrogen site location: inferred from neighbouring sites
*wR*(*F*^2^) = 0.101	H-atom parameters constrained
*S* = 1.26	*w* = 1/[σ^2^(*F*_o_^2^) + (0.036*P*)^2^ + 0.4143*P*] where *P* = (*F*_o_^2^ + 2*F*_c_^2^)/3
2075 reflections	(Δ/σ)_max_ < 0.001
83 parameters	Δρ_max_ = 0.70 e Å^−^^3^
0 restraints	Δρ_min_ = −0.55 e Å^−^^3^

### Special details

**Table:**

Geometry. All e.s.d.'s (except the e.s.d. in the dihedral angle between two l.s. planes) are estimated using the full covariance matrix. The cell e.s.d.'s are taken into account individually in the estimation of e.s.d.'s in distances, angles and torsion angles; correlations between e.s.d.'s in cell parameters are only used when they are defined by crystal symmetry. An approximate (isotropic) treatment of cell e.s.d.'s is used for estimating e.s.d.'s involving l.s. planes.
Refinement. Refinement of *F*^2^ against ALL reflections. The weighted *R*-factor *wR* and goodness of fit *S* are based on *F*^2^, conventional *R*-factors *R* are based on *F*, with *F* set to zero for negative *F*^2^. The threshold expression of *F*^2^ > σ(*F*^2^) is used only for calculating *R*-factors(gt) *etc*. and is not relevant to the choice of reflections for refinement. *R*-factors based on *F*^2^ are statistically about twice as large as those based on *F*, and *R*- factors based on ALL data will be even larger.

### Fractional atomic coordinates and isotropic or equivalent isotropic displacement parameters (Å^2^)

**Table:**

	*x*	*y*	*z*	*U*_iso_*/*U*_eq_	
C1	0.9103 (5)	1.0397 (3)	0.7493 (4)	0.0605 (7)	
H1A	0.8626	1.0161	0.8386	0.091*	
H1B	0.9085	1.1247	0.7405	0.091*	
H1C	1.0355	1.0117	0.7793	0.091*	
C2	0.7921 (3)	0.9875 (2)	0.5768 (3)	0.0415 (5)	
C3	0.6959 (4)	1.0566 (2)	0.4331 (4)	0.0525 (6)	
H3	0.7041	1.1389	0.4413	0.063*	
C4	0.5891 (4)	1.0046 (3)	0.2791 (4)	0.0524 (6)	
H4	0.5246	1.0512	0.1825	0.063*	
C5	0.5776 (3)	0.8820 (2)	0.2679 (3)	0.0424 (5)	
H5	0.5053	0.8449	0.1644	0.051*	
C6	0.6762 (3)	0.81599 (18)	0.4143 (3)	0.0317 (4)	
N1	0.7813 (2)	0.86822 (16)	0.5661 (2)	0.0330 (3)	
Cl1	1.20088 (11)	0.7500	0.88188 (13)	0.0521 (2)	
Cl2	0.74082 (14)	0.7500	0.94980 (13)	0.0511 (2)	
Zn1	0.89560 (5)	0.7500	0.76788 (4)	0.03392 (12)	

### Atomic displacement parameters (Å^2^)

**Table:**

	*U*^11^	*U*^22^	*U*^33^	*U*^12^	*U*^13^	*U*^23^
C1	0.0688 (19)	0.0401 (13)	0.0625 (17)	−0.0094 (12)	0.0104 (15)	−0.0154 (12)
C2	0.0457 (12)	0.0306 (10)	0.0480 (12)	−0.0039 (8)	0.0162 (10)	−0.0032 (8)
C3	0.0694 (17)	0.0281 (10)	0.0616 (16)	0.0033 (10)	0.0249 (14)	0.0063 (10)
C4	0.0641 (16)	0.0434 (13)	0.0473 (13)	0.0122 (12)	0.0168 (12)	0.0161 (11)
C5	0.0453 (12)	0.0424 (12)	0.0342 (10)	0.0048 (9)	0.0074 (9)	0.0057 (9)
C6	0.0326 (9)	0.0308 (9)	0.0300 (8)	0.0014 (7)	0.0091 (7)	0.0016 (7)
N1	0.0341 (8)	0.0288 (8)	0.0328 (8)	−0.0010 (6)	0.0075 (6)	−0.0002 (6)
Cl1	0.0325 (4)	0.0642 (6)	0.0492 (5)	0.000	0.0014 (3)	0.000
Cl2	0.0548 (5)	0.0567 (5)	0.0479 (4)	0.000	0.0255 (4)	0.000
Zn1	0.03172 (18)	0.03621 (19)	0.02830 (18)	0.000	0.00368 (12)	0.000

### Geometric parameters (Å, °)

**Table:**

C1—C2	1.499 (4)	C4—H4	0.9300
C1—H1A	0.9600	C5—C6	1.383 (3)
C1—H1B	0.9600	C5—H5	0.9300
C1—H1C	0.9600	C6—N1	1.350 (3)
C2—N1	1.347 (3)	C6—C6^i^	1.487 (4)
C2—C3	1.384 (4)	Zn1—N1	2.0569 (18)
C3—C4	1.366 (4)	Zn1—Cl1	2.2013 (11)
C3—H3	0.9300	Zn1—Cl2	2.2035 (10)
C4—C5	1.386 (4)	Zn1—N1^i^	2.0569 (18)
			
C2—C1—H1A	109.5	C6—C5—C4	118.4 (2)
C2—C1—H1B	109.5	C6—C5—H5	120.8
H1A—C1—H1B	109.5	C4—C5—H5	120.8
C2—C1—H1C	109.5	N1—C6—C5	121.6 (2)
H1A—C1—H1C	109.5	N1—C6—C6^i^	115.83 (11)
H1B—C1—H1C	109.5	C5—C6—C6^i^	122.51 (14)
N1—C2—C3	120.3 (2)	C2—N1—C6	119.82 (19)
N1—C2—C1	117.1 (2)	C2—N1—Zn1	126.50 (16)
C3—C2—C1	122.6 (2)	C6—N1—Zn1	113.51 (13)
C4—C3—C2	120.3 (2)	N1^i^—Zn1—N1	80.71 (10)
C4—C3—H3	119.8	N1^i^—Zn1—Cl1	115.45 (6)
C2—C3—H3	119.8	N1—Zn1—Cl1	115.45 (6)
C3—C4—C5	119.5 (2)	N1^i^—Zn1—Cl2	110.90 (6)
C3—C4—H4	120.3	N1—Zn1—Cl2	110.90 (6)
C5—C4—H4	120.3	Cl1—Zn1—Cl2	117.76 (5)
			
N1—C2—C3—C4	0.0 (4)	C5—C6—N1—C2	−0.2 (3)
C1—C2—C3—C4	−179.6 (3)	C6^i^—C6—N1—C2	178.78 (16)
C2—C3—C4—C5	0.1 (5)	C5—C6—N1—Zn1	175.34 (17)
C3—C4—C5—C6	−0.2 (4)	C6^i^—C6—N1—Zn1	−5.7 (3)
C4—C5—C6—N1	0.3 (4)	C2—N1—Zn1—N1^i^	−178.10 (16)
C4—C5—C6—C6^i^	−178.63 (19)	C6—N1—Zn1—N1^i^	6.69 (17)
C3—C2—N1—C6	0.0 (4)	C2—N1—Zn1—Cl1	−64.2 (2)
C1—C2—N1—C6	179.7 (2)	C6—N1—Zn1—Cl1	120.55 (13)
C3—C2—N1—Zn1	−174.88 (19)	C2—N1—Zn1—Cl2	73.0 (2)
C1—C2—N1—Zn1	4.7 (3)	C6—N1—Zn1—Cl2	−102.24 (14)

Symmetry codes: (i) *x*, −*y*+3/2, *z*.
